# Barriers and facilitators of self‐management of diabetes amongst people experiencing socioeconomic deprivation: A systematic review and qualitative synthesis

**DOI:** 10.1111/hex.14070

**Published:** 2024-05-15

**Authors:** Abi Woodward, Kate Walters, Nathan Davies, Danielle Nimmons, Joanne Protheroe, Carolyn A. Chew‐Graham, Fiona Stevenson, Megan Armstrong

**Affiliations:** ^1^ Research Department of Primary Care and Population Health University College London London UK; ^2^ School of Medicine Keele University Keele UK; ^3^ Wolfson Institute of Population Health Queen Mary University of London London UK

**Keywords:** diabetes, health inequalities, long‐term condition, qualitatvie, self‐management, socioeconomic deprivation

## Abstract

**Background:**

The number of people living with diabetes is rising worldwide and a higher prevalence of diabetes has been linked to those experiencing socioeconomic deprivation. Self‐management strategies are vital and known to reduce the risks of long‐term complications amongst people living with diabetes. Lack of knowledge about self‐care activity required to manage diabetes is a key barrier to successful self‐management. Self‐management interventions can be less effective in socioeconomically deprived populations which can increase the risk of exacerbating health inequalities. The purpose of this review is to identify and synthesise qualitative evidence on the barriers and facilitators of self‐management of diabetes amongst people who are socioeconomically disadvantaged.

**Methods:**

MEDLINE, EMBASE, AMED, PsycINFO and CINAHL Plus were searched for qualitative studies concerning self‐management of multiple long‐term conditions amongst socioeconomically disadvantaged populations. Relevant papers which focused on diabetes were identified. Data were coded and thematically synthesised using NVivo.

**Findings:**

From the search results, 79 qualitative studies were identified after full‐text screening and 26 studies were included in the final thematic analysis. Two overarching analytical themes were identified alongside a set of subthemes: (1) Socioeconomic barriers to diabetes self‐management; healthcare costs, financial costs of healthy eating, cultural influences, living in areas of deprivation, competing priorities and time constraints, health literacy, (2) facilitators of diabetes self‐management; lifestyle and having goals, support from healthcare providers, informal support.

**Discussion:**

Self‐management of diabetes is challenging for people experiencing socioeconomic deprivation due to barriers associated with living in areas of deprivation and financial barriers surrounding healthcare, medication and healthy food. Support from healthcare providers can facilitate self‐management, and it is important that people with diabetes have access to interventions that are designed to be inclusive from a cultural perspective as well as affordable.

**Patient or Public Contribution:**

A patient advisory group contributed to the research questions and interpretation of the qualitative findings by reflecting on the themes developed.

## 1 INTRODUCTION

One in 11 adults worldwide is living with diabetes^1^; 90% of whom have type 2 diabetes.^2^ Within the United Kingdom, over 4.9 million people are living with diabetes, both diagnosed and undiagnosed; a figure that is set to rise to 5.3 million by 2025.^3^ Diabetes is caused by a loss of the physical or functional β‐cell mass, mostly due to an autoimmune process (type 1 aetiological process) and/or increased need for insulin due to insulin resistance (type 2 process).^4^ Experiencing socioeconomic deprivation has been linked to higher prevalence of type 1 and 2 diabetes and is shown to disproportionately effect low‐income adult populations and ethnic minorities.^5^ Socioeconomic deprivation includes a range of interconnected characteristics that impact upon inequalities and disadvantage.^6^ For example, living in a socioeconomically disadvantaged area is shown to be characterised by detrimental lifestyle factors throughout the life course which impacts negatively on health outcomes. The relative risk of diabetes is, therefore, almost four times higher for people with high cumulative neighbourhood socioeconomic disadvantage compared to those with low disadvantage.^7^

Research suggests that substantial system‐level improvements are needed with regards to the quality of diabetes care.^8^ Supporting people in managing their long‐term conditions is connected with improved health outcomes, in a variety of conditions.^9^ Self‐management refers to an individual's ability to manage the symptoms, treatment and psychological impacts and lifestyle changes inherent in living with chronic conditions such as diabetes.^10^ Self‐management requires taking a proactive approach to managing health conditions such as through accessing preventative services.^11^ Self‐management of diabetes is known to reduce the risks of long‐term complications and is associated with various individual factors that can either impede or promote good self‐management.^12^, ^13^ Furthermore, evidence of self‐management in socioeconomic deprivation, specific to multiple long‐term conditions, found that greater awareness is needed amongst health professionals of the barriers/challenges of self‐management.^14^, ^15^

One of the key barriers to the management of diabetes relates to a lack of knowledge about self‐care which can increase nonadherence of activities relating to diet, exercise, blood glucose monitoring and foot care.^12^ A report published in 2021 suggests that better management and prevention of conditions such as diabetes, which are at the centre of disease clusters (i.e., a greater number of cases of a disease than expected within a group of people in a geographical area in a specific time period) and potentially part of several other chronic conditions' trajectories, would improve health outcomes.^16^ However, self‐management interventions can be less effective in socioeconomically deprived populations and therefore run the risk of exacerbating health inequalities.^17^

The aim of this review is to identify and synthesise evidence on the barriers and facilitators of self‐management of diabetes (type 1 and 2) amongst people who are socioeconomically disadvantaged and explore how self‐management can be optimised in this population.

## 2 METHODS

This systematic review uses a thematic synthesis methodology and was selected based on the descriptive nature of qualitative studies.^18^ The review is informed by ENTREQ guidelines^19^ and reported according to the Preferred Reporting Items for Systematic Reviews and Meta‐Analysis (PRISMA)‐equity guidelines^20^ and guidance for thematic synthesis.^18^ The review protocol is registered on the PROSPERO database (8 November 2021 CRD42021289674). Available from: https://www.crd.york.ac.uk/prospero/display_record.php?ID=CRD42021289674.

### 2.1 Inclusion and exclusion criteria

Studies were included if they:

1. Used qualitative methods in their approach to data collection and analysis.
2. Included adults over 18 years of age with diabetes who were experiencing socioeconomic deprivation (with a proxy or quantifiable measure, e.g., low income, low income or from an area of deprivation).
3. Explored the self‐management of diabetes (type 1, 2 or both).

Studies were excluded if:

1. Data could not be separated to identify the perspectives of those experiencing socioeconomic deprivation.
2. The full text was not available to obtain in English.
3. The papers were review articles, editorials or conference proceedings.

### 2.2 Search strategy

Database searches were conducted in MEDLINE, EMBASE, AMED, PsycINFO and CINAHL Plus. Databases were originally searched to identify any long‐term condition due to the initial focus of another systematic review on self‐management.^14^ Key terms and Medical Subject Headings of self‐management and variations of the terms ‘long‐term conditions’ (including ‘diabetes’) and ‘socioeconomic deprivation’ (e.g., socioeconomic status/position) were included in the search, without date or language restrictions. Please see Table S1, for example, search strategy. Screening of abstracts and titles was conducted independently by two authors (A. W. and M. A.) and papers that explored diabetes and self‐management qualitatively were separated out. Full‐text papers were screened by the first reviewer (A. W.), and all were checked by a second reviewer (M. A.). The eligibility of the papers and any discrepancies was discussed with the wider review team (K. W., N. D., D. N., J. P., C. A. C.‐G., F. S.).

### 2.3 Quality assessment

A quality assessment of the literature was carried out using the Critical Appraisal Skills Programme (CASP) tool.^21^ This checklist consists of 10 questions which look at the results of the studies, their validity (i.e., suitability of methodological approaches used to obtain them), and how valuable and/or transferral the results are. Studies were reviewed based on the results of the quality assessment, but no studies were excluded based on quality. Utilising the CASP method highlighted the range in quality of the studies and whether recruitment and data analysis techniques were appropriate. These factors have been taken into consideration during the presentation of results.

### 2.4 Thematic synthesis

The articles were analysed using thematic synthesis, which involves three stages: the coding of text line‐by‐line; the development of descriptive themes; and the generation of analytical themes.^18^ Each article was imported into NVivo (Release 1.7.1) software by A. W. (a health researcher with a background in sociology and health inequalities) who coded each line of text in the results sections. Descriptive themes were developed around the self‐management (as defined previously) of diabetes, and the barriers and facilitators to self‐management amongst people experiencing socioeconomic deprivation by A. W. and M. A. (a health researcher with a background in psychology and health inequalities). The themes were developed further into analytical with the wider review team (K. W., N. D., D. N., J. P., C. A. C.‐G., F. S.) who include clinical and nonclinical academics with experiences in self‐management, primary care, healthy ageing and inequalities, as well as our two patient and public involvement members. Themes moved from descriptive to analytical by exploring the interpretation and context of our findings in this population and receiving feedback from people experiencing socioeconomic deprivation and those who work with them.

## 3 RESULTS

**Figure 1:**
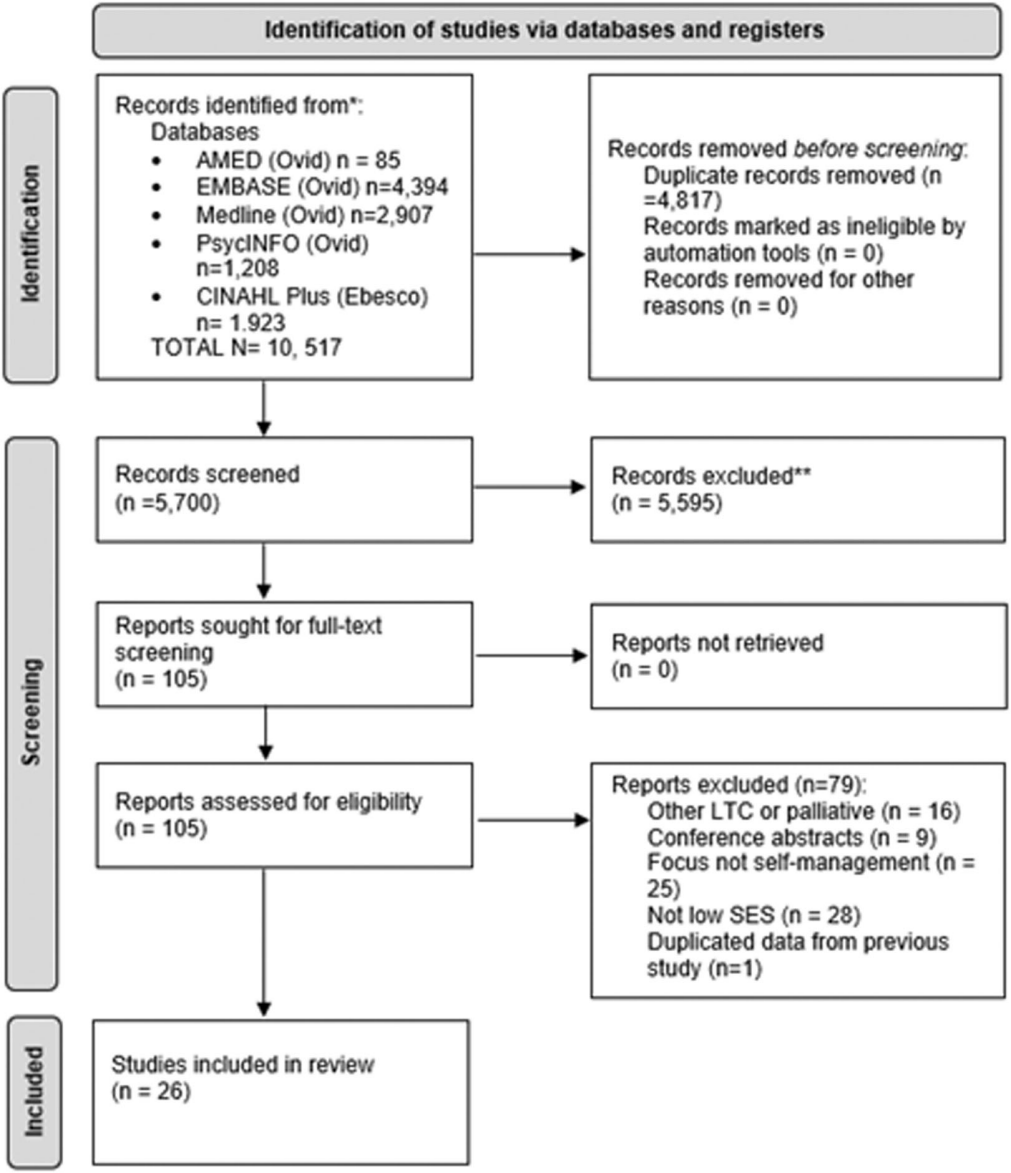
Preferred Reporting Items for Systematic Reviews and Meta‐Analysis flow diagram. *Consider, if feasible to do so, reporting the number of records identified from each database or register searched (rather than the total number across all databases/registers). **If automation tools were used, indicate how many records were excluded by a human and how many were excluded by automation tools. From: Page et al.^22^ For more information, visit: http://www.prisma-statement.org. LTC, long‐term care; SES, socioeconomic status.

The PRISMA flow diagram (Figure 1) relates to the diabetes records, although the initial search was conducted on all long‐term conditions.

### 3.1 Study characteristics

All the studies included participant samples that had characteristics associated with socioeconomic deprivation. These were most typically characterised through low‐income, low educational attainment, living in an area of deprivation and experiences of homelessness. There were variances surrounding how levels of socioeconomic deprivation were determined by different authors, but clear definitions or measures were rarely provided. The studies were conducted in Australia (*n* = 3), Canada (*n* = 3), Denmark (*n* = 1), Mexico (*n* = 1), Sweden (*n* = 1) and United States (*n* = 17). Five of the 26 studies included were process evaluations.^23^, ^24^, ^25^, ^26^, ^27^

The included studies largely relate to individuals diagnosed with type 2 diabetes, with two of these also specifying type 1 and type 2.^25^, ^28^ Six studies^23^, ^24^, ^27^, ^29^, ^30^, ^31^ specified that individuals must be diagnosed with diabetes but did not provide details on what type. Further details relating to participant characteristics are presented in Table 1.

**Table 1:** Primary study characteristics (*n* = 26).

Reference, country	Aim	Condition detail	Study population	Socioeconomic characteristics of sample	Analysis method	Key findings
Akohhoue et al.,^32^ United States	To explore strategies to improve self‐management of type 2 diabetes mellitus (T2DM) amongst low‐income and minority groups.	Type 2 diabetes (T2D)(diagnosed for at least 1 year).	17 patients, aged 21 or older 5 caregivers (3 women, 2 men), aged 28–54 15 healthcare providers (9 women, 6 men), aged 29–54 Ethnicity: unknown.	Authors stated that 53% had low income (below $15,000) and 71% had low educational attainment. All were patients from a private family clinic.	Method unclear (implementation guide was used to guide the analysis of focus group transcripts).	Most patients had less than optimal self‐care behaviours. Patient and caregiver strategies include improving patient–provider communication, provider accessibility and compassion and flexible clinic hours.
Allen et al.,^23^ United States	To identify factors influencing participant engagement in a community‐based diabetes self‐management programme with a focus on the needs of underserved groups.	Participants diagnosed with diabetes and enroled in Diabetes Control Programme.	22 participants (14 women, 8 men), aged 45–76 Ethnicity: unknown.	Authors stated that participants were recruited from a Diabetes Control Programme to focus on those typically underserved by clinic‐based diabetes self‐management programmes. No further categorisation of socioeconomic deprivation provided.	Rigorous and accelerated data reduction.	Fear affected enrolment and retention. Peers and coaches were important for social support and accountability. The length of the programme, accessible information, practical skill building, emphasis on small improvements to achieve larger goals were critical for engagement and improving self‐management.
Aweko et al.,^33^ Sweden	To explore patient and provider perceptions and experiences of T2D self‐management.	T2D.	12 patients (6 women, 6 men), aged 35–60 Ethnicity: Asian, East and West African 10 healthcare providers (8 women, 2 men) Ethnicity: White, African, South American, Middle Eastern.	Authors stated that patients lived in communities with low‐income levels, high unemployment and generally poor housing (according to the Care Need Index).	Thematic analysis.	Patients found it difficult to tailor information and lifestyle advice to fit their daily life. Healthcare providers recognised that patients needed support to change behaviour but saw themselves as inadequately equipped to deal with the different cultural and social aspects of self‐management.
Burner et al.,^24^ United States	To examine amongst low‐income, urban Latinos with diabetes, the effect of a mobile health intervention designed to increase knowledge, self‐efficacy and subsequent disease management and glycaemic control.	Participants diagnosed with diabetes.	24 participants (16 women, 8 men), aged 26–76 Ethnicity: Latino (18) and non‐Latino (6).	Authors stated that participants were low‐income, and the majority were uninsured (92%).	Grounded theory approach.	Participants believed TExT‐MED improved their diabetes management. Strengths included messages cueing specific behaviours such as medication reminders and challenge messages.
Campbell et al.,^34^ Canada	To explore diabetes and homelessness through a community‐based participatory research approach.	T2D.	8 participants (5 women, 3 men), aged under 45–65 Ethnicity: White (5) and unknown (3).	Authors stated that participants were either homeless, unstably housed or in temporary housing.	Inductive thematic analysis	Homelessness impacted emotional and mental health, impairing the ability to focus on diabetes self‐management. Barriers faced included unhealthy food in vending machines at shelters, unpalatable meals, challenges accessing health services and adhering to medication regimens when homeless.
Carolan et al.,^35^ Australia	To explore the experiences and concerns of individuals with T2DM, in a predominantly low socioeconomic setting.	T2D	22 participants, aged 40 to more than 70 Ethnicity: unknown.	Authors stated that participants lived in a predominantly low socioeconomic area by Australian standards. No further clarification provided.	Thematic analysis.	Overall, the impact of diabetes on the family, and the importance of family members in providing support and encouragement to assist their self‐management efforts is highlighted.
Chan et al.,^28^ Canada	To explore how food insecurity affects individuals' ability to manage their diabetes.	Diagnosed with either type 1 diabetes or T2D.	21 participants (10 women, 11 men), aged 20–70 Ethnicity: Caucasian (12), Caribbean (5), African (2) and Middle Eastern (2).	Authors states that participants were part of a low‐income population. Those included had experience of food insecurity in the past year. One‐third had high school education or less.	Thematic analysis.	People with diabetes who are food insecure experienced barriers to preparing and accessing appropriate food, social isolation, enhancing agency and improving resilience.
Christensen et al.,^36^ Denmark	To explore barriers and facilitators related to self‐management in vulnerable patients with T2D, and healthcare professionals.	T2D	12 patients (8 women, 4 men) 3 family members 16 healthcare professionals (female), aged 37–63 Ethnicity: unknown.	Authors stated that the catchment area for recruitment is characterised by a high proportion of Urdu‐speaking ethnic minorities, low income and educational levels, and high unemployment rates compared to the rest of the Capital Region.	Systematic text condensation	Patient barriers to diabetes self‐management (DSM) included lack of access to DSM support, judgement from one's social environment, feeling powerless. Patient facilitators included a person‐centred approach, peer support and practical knowledge about DSM. Healthcare professionals recognised the need to approach patients differently.
Clark et al.,^37^ United States	To explore the political and economic contexts that shape the experience of diabetes for Mexican American adults, using components of explanatory models from people with diabetes and their family caregivers.	T2D (diagnosed for at least 1 year).	20 patients (15 women, 5 men), aged 27–73 Ethnicity: Mexican American 20 caregivers (12 women, 8 male), aged 20–68 Ethnicity: largely Mexican American.	Authors stated that the research was conducted in a low‐income suburb and a high proportion of participants were reported to be on a low‐income (<20,000 USD).	Critical ethnography.	DSM is tied to other mental and bodily states. Family and neighbourhood environments cause stress and prevent diabetes solutions. The hassles of the health care environment subvert self‐management.
Dao et al.,^38^ Australia	To investigate the factors influencing self‐management of T2D (T2DM) in general practice.	T2D (poorly controlled)	23 participants (10 postintervention patients, 13 providers). Ethnicity: unknown.	Authors stated that participants were recruited from an area with a higher rate of socioeconomic disadvantage than the rest of Sydney.	Thematic analysis.	Factors influencing self‐management include motivation, time constraints, e‐health literacy, patient–provider relationship, availability and affordability of multidisciplinary care, and cultural barriers to maintaining a healthy diet.
Fritz et al.,^39^ United States	To examine the process by which low‐income women develop the skills to integrate diabetes self‐management into daily life and the conditions that affect the process.	T2D (self‐identified diagnosis).	10 women, aged 40–64 Ethnicity: Black (7) and White (3).	Authors stated that participants were low income (the income range for participants was <5000–29,000 USD). Low income was defined as having a total household income at or below 200% of the federal poverty guidelines.	Grounded theory approach.	Inquiry and practice play key roles in assimilating DSM. By practicing, participants developed strategies to facilitate consistent engagement in DSM components. Participants were more likely to act on information that came from a supportive provider.
Gazmararian et al.,^29^ United States	To explore individual, educational and system barriers that limit low‐income diabetes patients' ability to achieve optimal DSM.	Diagnosed with diabetes.	35 patients Ethnicity: African American (31), White (3) and Latino (1).	Authors stated that participants were recruited from a severely economically disadvantaged population. Approximately a third had less than high school level reading skills and 40% were not currently working.	Method unclear	Denial was a key factor that inhibited adherence to a healthy mode of living. Educational barriers were failure to recognise risks and consequences of an asymptomatic condition.
Henderson et al.,^40^ Australia	To explore access to capital in the form of economic resources, social networks and health knowledge to successfully negotiate and undertake self‐management activities.	T2D.	28 participants (14 women, 14 men), aged 32–76 Ethnicity: unknown.	Authors stated that participants were from a lower socioeconomic region of Adelaide. Majority had a high school education or trade education (*n* = 26).	Exploratory qualitative approach.	Access to capital is a significant barrier to DSM. Participants often lacked economic and social capital in the form of health‐promoting support networks and engaged in social network activities that conflicted self‐management, disadvantaging their relationships with health professionals.
Hu et al.,^41^ United States	To explore the barriers to DSM from the perspective of Hispanic immigrants with diabetes and their family members.	T2D.	36 patients and 37 family members (75% female), average age of 50 Ethnicity: Hispanic.	Authors stated that participants were recruited from a clinic providing services to a large population of low‐income Hispanic people. Majority were immigrants from Mexico (77.8%).	Thematic content analysis.	Barriers to self‐management included physically/emotionally suffering from diabetes, difficulty in managing the disease via medication and dietary requirements and lack of resources/support from the health care system and family members. Family members could support but felt they lacked knowledge.
Keene et al.,^42^ United States	To examine how housing challenges and housing resources shape DSM behaviours.	T2D.	40 participants (19 women, 21 men), average age of 51 Ethnicity: Black (25), White (7), Hispanic/Latino (3) and multiracial/other (5).	Authors stated that participants were low income with 50% living in rent subsidised housing and 15% homeless. Most were in receipt of disability and/or other state benefits.	Grounded theory approach.	Instability in housing access affected participants' ability to prioritise diabetes care, establish and maintain diabetes routines and afford diabetes‐related expenses.
Luo and White‐Means,^25^ United States	To investigate the willingness to use diabetes apps in patients with limited access to primary care providers.	Participants diagnosed with type 1 diabetes or T2D.	15 patients Ethnicity: unknown.	Authors stated that participants were recruited from an area which has a predominant minority residency and underserved characteristics.	Interpretive phenomenological analysis.	Patients were willing to try at least one diabetes‐related app, despite limited experience with technology. App functions should be individualised to each patient's needs for maximum benefit. Barriers included knowledge and technical challenges, and security issues.
Lynch et al.,^43^ United States	To explore low‐income minority patients' concepts of DSM and assess the extent to which patient beliefs correspond to evidence‐based recommendations.	T2D.	84 participants, average age of 55 for Mexican American (43% female) and 57.6 for African American (48% female) Ethnicity: Mexican American (49) and African American (35).	Authors stated that most participants either met financial eligibility for federal insurance or were unable to afford insurance. The majority of Mexican American participants had less than high school education (71%).	Grounded theory approach.	Strategies for diabetes self‐care were medication use, diet, weight loss and exercise. African Americans expressed scepticism about taking medications. Mexican Americans faced barriers acquiring medication. Blood glucose self‐monitoring and reducing risks of diabetes complications were rarely mentioned.
Mamykina et al.,^26^ United States	To investigate subjective experiences and patterns of engagement with novel electronic tool for facilitating reflection and problem solving for individuals with T2D.	Diagnosed with T2D mellitus and a glycated haemoglobin level equal to or greater than 8.0.	15 participants (12 women, 3 men), aged 25–63 Ethnicity: African American (10), Hispanic (4) and unknown (1).	Authors stated that participants were clients of community health centres that deliver care to socioeconomically disadvantaged populations and ethnic minorities.	Inductive bottom‐up thematic analysis.	Individuals used MoDD to identify problematic blood glucose patterns, explore behavioural triggers contributing to patterns, selecting alternative behaviours, implementing behaviours while monitoring for improvements.
Onwudiwe et al.,^30^ United States	To explore patients' perceptions about barriers to DSM and associations with poor health outcomes amongst minority patients.	Diagnosed with diabetes.	31 patients (majority female), aged 43–81 Ethnicity: predominantly African American.	Authors stated that the sample was a low‐income, minority group. No further details are provided.	Unknown	Participants found some diabetes health information to be confusing. Physicians aren't forthcoming with information relating to diabetes. A lack of awareness of target blood glucose and blood pressure goal was acknowledged by a majority of participants.
Pilkington et al.,^44^ Canada	To enhance understanding about how living on a low income affects patients' self‐management of T2D, from their perspective.	T2D.	60 participants (34 women, 26 men), aged 30–82 years Ethnicity: unknown.	Authors stated that participants were all low‐income. The majority (57.6%) were reported as receiving their primary source of income from welfare/disability support.	Thematic analysis.	Participants had to balance DSM and competing priorities like daily survival. Management of diabetes was assisted by social supports and health services. Participants had a basic knowledge about diabetes management.
Ramal et al.,^45^ United States	To explore the lived experience and identify factors that impact DSM for limited English‐speaking Hispanics with T2D living in low socioeconomic status neighbourhoods.	Participants with T2D or family members of people with diabetes.	27 participants (21 women, 6 men), aged 45–60 Ethnicity: Hispanic	Authors stated that participants were from a predominantly low socioeconomic, Hispanic neighbourhood. Majority were unemployed (*n* = 24).	Grounded theory approach.	Access to resources, struggles with diet, self‐efficacy, social support and the underlying pervasive influence of the family has the potential to enhance/limit self‐management of diabetes.
Reyes et al.,^46^ United States	To explore barriers and facilitators for DSM in underserved adults with T2D.	T2D.	44 patients (30 women, 14 men), mean age of 55.3 Ethnicity: African American (9), Caucasian (29), Latino (4), Native American (1) and other (1).	Authors stated participants were from an underserved population comprising of patients from Federally‐Qualified Health Centres (vanguard providers of underserved patients). One quarter of participants (25%) did not complete high school and 18% were unemployed.	Thematic analysis.	Patients with uncontrolled and controlled glycaemic control described the impact of diet restriction on social interactions, impact of mental health on self‐management, formal/informal support and lack of support from providers. Uncontrolled patients experienced confusion whereas controlled felt positive about self‐management.
Shepherd‐Banigan et al.,^27^ United States	To describe participant experiences of a household‐level, community health worker led intervention to improve diabetes‐related health behaviours and outcomes.	Diagnosed with diabetes.	40 participants (32 women, 8 men), aged 26–83 Ethnicity: Hispanic.	Authors stated that more than half of participants had low educational attainment and 40% were on Medicaid, Medicare or a basic health care insurance.	Thematic analysis.	*Promotores* (community health workers) were important for participants as they provided social support and encouraged behaviour change. Tools providing step‐by‐step examples of healthy behaviours or aiding monitoring of behaviour change serve to build skills and confidence to achieve goals.
Stotz et al.,^47^ United States	To explore patient perspectives on socioeconomic barriers related to DSM and interventions to address these barriers.	T2D.	53 participants (23 women, 30 men), aged 33–84 Ethnicity: Hispanic/Latinx (6), White (37), Black (8) and Asian/other/multiple (2).	Authors stated that participants were recruited from practices that have socioeconomic diversity. 43% had Medicaid health insurance and the majority had high school level education only (56%).	Constant comparison method framed by constructivist theory.	Participants found existing nutrition resources insufficient to support health eating for diabetes. Socioeconomic circumstances make health eating challenging for diabetes management. Participants indicated a desire for interventions to address socioeconomic barriers.
Vest et al.,^31^ United States	To examine how access to social capital amongst low‐income populations facilitates and impedes their self‐efficacy in DSM.	Diagnosed with diabetes for at least 1 year.	34 patients (26 women, 8 men), mean age of 58 Ethnicity: African (6), Asian (5), Latino (11), White (6), African American (4) and Native American (2).	Authors stated that all participants were recruited from a low‐income neighbourhood which is amongst one of the poorest sections of the city (according to census tracts).	Content‐driven immersion‐crystallisation approach.	Patients' ability to self‐manage diabetes is influenced by social support networks, the doctor–patient relationship and interaction with the health care system. Access to social capital that would increase self‐efficacy in diabetes management is tied to social relationships and their access to resources.
Whittemore et al.,^48^ Mexico	To identify the challenges amongst adults with T2D self‐management and perceptions of healthcare providers.	T2D (diagnosed for at least 1 year).	20 participants (16 women, 4 men), mean age of 52.5 Ethnicity: Unknown. 19 providers (15 women, 4 men), mean age of 41.6.	Authors stated that the participants included in the study were ‘socioeconomically vulnerable’. 55% had preschool or primary school as their highest level of education. 70% were reported to have only enough money to meet basic needs.	Content analysis.	Personal challenges for DSM included cultural beliefs, lack of resources, challenges to lifestyle modification, lack of family support/competing demands and mental health issues. System level challenges included lack of resources, perceived quality of care and patient engagement barriers.

### 3.2 Quality assessment

The CASP tool highlighted that several studies lacked detail about whether the relationship between researcher and participants had been adequately considered.^30^, ^42^, ^47^ The quality assessment highlighted some issues due to insufficient information, resulting in a ‘can't tell’ score which was the case for two studies in relation to the data analysis process.^30^, ^32^ One paper^32^ scored ‘No’ for the question relating to a clear statement of findings. While there was a clear section within the paper, the authors did not present enough detail relating to the qualitative results. See Table S2 for full quality assessment of the studies. The data themes were assessed using the Confidence in the Evidence from Reviews of Qualitative research (CerQual). Following the CerQual guidelines,^48^ the confidence was high to moderate (see Table S3).

### 3.3 Data synthesis results

The data presented in this section were generated from a set of analytical themes as highlighted in Table 2.

**Table 2:** Relationship between analytical themes and subthemes.

Analytical themes	Subthemes
Socioeconomic barriers to diabetes self‐management	Healthcare costs
	Financial costs of healthy eating
	Cultural influences
	Living in areas of deprivation
	Competing priorities and time constraints
	Health literacy
Facilitators of diabetes self‐management	Lifestyle and goals
	Support from healthcare providers
	Informal support

### 3.4 Socioeconomic barriers to diabetes self‐management

#### 3.4.1 Healthcare costs

In 10 studies,^31^, ^33^, ^36^, ^39^, ^40^, ^41^, ^42^, ^44^, ^46^, ^49^ financial instability impacted medication adherence because of difficulties affording medications. This is demonstrated below by two Mexican American participants from different studies:

Because that's why I quit checking myself—I couldn't afford that stuff [test strips and glucose monitor], it was too expensive.^36^ ^,p.390^ (United States)

But even with medication the thing of it is … down there where I live I don't take my medicine the way I should because I don't have enough medication. Like a 30‐day bottle I'll try to prolong it for a couple of months or three months.^49^ ^,p.818^ (United States)

The second quote above connects with the challenges that many US‐based participants faced around the high cost of medication, a lack of medical insurance and difficulties navigating the healthcare system.^49^ The issues of having enough money to afford the cost of prescriptions was also highlighted in three further studies.^33^, ^42^, ^44^ One participant who was homeless, was faced with paying $97.14 for a 1‐week supply of medication:

I have to decide if I eat this week, or if I refill my prescriptions.^33^,p.1039 (Canada)

Another participant who had private insurance explained the challenges she still faced in relation to affording medication and supplies:

I haven't taken my blood sugar at home in ages. The strips cost $30 on the co‐pay. I cannot afford that. My insulin is $50. I can't afford it. I cannot afford it. I've got insurance … but I can't afford those strips … It's an expensive disease.^31^ ^,p.152^ (United States)

The above participant was reported to have periods without insurance coverage or care because of the costly fees and premiums involved. Another participant, who had insurance through Medicare, which is a US federal programme for older and disabled adults said:

There are medications I can't get because I can't afford them. When I'm done paying my rent and making sure my son has food, I don't have extra $10. I don't. I have just enough to pay my bills.^41^ ^,p.75^ (United States)

In this instance, the above participant could not manage healthcare payments (medication fee) through Medicare alongside her rent and other financial demands. Others in the same study, however, were said to have ‘virtually no healthcare expenses as a result of their coverage through Medicaid’ which is a US federal and state programme providing healthcare coverage to those on a low‐income.^41^ The study indicates some discrepancies across health insurance coverage for Americans experiencing socioeconomic deprivation, highlighting that the ability to engage in diabetes self‐management is broadly impacted by the US health system.

#### 3.4.2 Financial costs of healthy eating

Living on a limited income impacted diet adherence which was needed to aid diabetes self‐management, and difficulties around the affordability of healthy food were highlighted in 12 studies. Two participants who received food stamps discussed the challenge of affording food:

I am on a fixed income and I only get so many food stamps and the [diabetic] cookbook, you can't afford that stuff in that cookbook.^44^ ^,p.7^ (United States)

…I get food stamps. They give me $166 a month. And that's not enough for all the stuff that you need to buy.^45^ ^,p.156^ (United States)

Some participants admitted that they turned to cheaper foods which were not suitable for facilitating diabetes self‐management but as the second quote below demonstrates, it could be difficult to make money stretch:

I don't have a lot of money … so I'll buy junk food, instead of real food, because the junk food is cheaper.^36^ ^,p.4^ (United States)

I look which [food] is cheaper because my money is very small, so after giving rent, I just have little bit money … Sometimes after the 20th [of the month] my money finished. It's very tight … So then I buy rice, because it's the main food … But when I eat [cheap rice] my sugar is going up … Sometimes I borrow money because I have to be conscious about my health.^42^ ^,p.124^ (Canada)

In addition, financial constraints around food could be compounded by issues such as social isolation. Some participants reported finding social situations difficult due to dietary requirements^32^ but also that limited finances impacted on going out.^28^ One participant explained how they worked to combat their experience of loneliness:

I think mostly it's the social factor of eating alone. I don't like eating alone. So …there's a community dinner that I go to, my [church group] … I've gone there for years … I go to drop‐ins with my friends for two reasons: one, because I like to eat with other people, and two, because I can't afford to buy food anymore … some of the places have really good food. Some of them have food that's very high in carbohydrates, which isn't good for diabetics.^28^ ^,p.5^ (Canada)

As the above extract demonstrates, accessing a community dinner assisted the participant with the cost of food. The quote is a further example of how people with diabetes, who also experience socioeconomic deprivation, have reduced control over their food choices to support diabetes self‐management.

#### 3.4.3 Cultural influences

Cultural factors at home and within families could act as a barrier to diabetes self‐management, especially around diet adherence. One participant explained that it was difficult to avoid specific foods as advised by their dietitian, suggesting that more awareness is needed around cultural norms:

I went to see the diabetic nurse and a dietician and I have endocrinologist. The main thing they tell me: ‘Cut the carbohydrates. Don't eat rice or potatoes’. But it's hard to do without rice, because we [ethnic group] generally eat rice every day…^42^,^p.123^ (Canada)

Another participant highlighted how the dietary advice they were given was not in line with the food types they would eat:

We [people of a similar culture] don't mess with stuff like quinoa, or however you call it. Couscous? Please! I mean, a whole grain to me is wheat bread. I can't see messing with some of that other stuff. I mean, I can't even say what it's called [pronounce it].^38^ ^,p.880^ (United States)

Several studies reported that access to local amenities to buy healthy food was challenging. One participant with limited transport options said:

The last one [dietician] I saw wanted me to have all sorts of things that I couldn't even find in the supermarket.^39^ ^,p.339^ (Australia)

The same study highlighted aspects of ‘working class masculinity’ in relation to excessive alcohol consumption. As such, it could be the case amongst some that individual health behaviours were influenced by the people they spent leisure time with, as illustrated in the quote below:

… when the son‐in‐law comes round he doesn't do anything else but drink [alcohol]. He's 40 and he's—if instead of having a cup of tea or coffee he'll have something out the fridge so I'll have three or four with him.^39^ ^,p.343^ (Australia)

#### 3.4.4 Living in areas of deprivation

The areas in which some participants lived limited the amount of physical activity they did due to not feeling safe:

This area is not actually conducive to walking, I don't feel that safe. I walk around to the shop, but I don't do the walking that I used to do.^39^ ^,p.340^ (Australia)

Some participants also reported that access to local services to facilitate self‐management were limited within the area they lived:

I have asked about an exercise class last time I was there [at the GP] and then I was told that it is the municipality that must take care of that. Then you are supposed to contact the municipality, which is quite difficult. Maybe other people find it easier, but I have not been able to get through with anything in the municipality.^35^ ^,pp.572–573^ (Denmark)

Insecure or precarious housing, which is associated with socioeconomic deprivation, also impacted on self‐management. Three studies^33^, ^41^, ^42^ highlighted how participants who relied on homeless shelters had limited food choices which led to poor control over diet:

I think it's more harder when I was homeless because I said kitchen, the shelters, they feed you pasta. And if you out there all day and didn't eat nothing, you eat whatever they give you.^41^ ^,p.75^ (United States)

#### 3.4.5 Competing priorities and time constraints

In 15 of the studies, there were competing priorities that participants with diabetes experienced in daily life, and in the longer‐term, meaning management of their diabetes was not always a priority. For those that had caring roles within families, the needs of others often took priority, impacting on the time that individuals had for their own self‐care:

Exercise, yeah I work full‐time and having, yeah, a disabled daughter it's just time, time's just‐ time.^37^ ^,p.179^ (Australia)

My daughter is very sick. She cannot do anything for herself. I haven't done anything for myself … I get very hungry and I eat the same thing I give her because I do not have time to cook for myself. I know that's bad for me, but I cannot take care of myself the way I should be.^44^ ^,p.7^ (United States)

In addition, several studies^29^, ^37^, ^38^, ^41^, ^42^, ^45^, ^46^ found that participants were unable to prioritise diabetes self‐management due to their employment situation. One participant spoke about the impact that his diabetes could potentially have on his ability to work if he was to take his insulin. This had left him in a precarious position:

My doctor told me my A1C [blood sugar levels] was 10.5, and that I was going to be on insulin. But I'm not going to take it. I drive a truck for a living and my boss won't let me drive if I'm on insulin. I have 4 kids, so I have to work.^29^ ^,p.783^ (United States)

Other participants found it challenging to manage their condition around the competing demands of their working lives:

…I work at night, office cleaning. I arrive home around 11:30 PM, I fall to sleep between 3:00 and 4:00 AM, wake up at 10:30 AM … that's every single day … Now my sugar levels are always higher [rather] than lower … and it makes me feel worried, because I don't want to have problems with my vision or anything like that … I don't [test my blood] every day; 3 days a week, because the strips are very expensive … Sometimes I don't take the medicine every day, because the medicine is expensive and my husband is not working, and because it is not the only medicine that I have to take, so I try to make it last.^42^ ^,p.122^ (Canada)

You do a 12, 13, 14 hour day you start at like 4, 5 in the morning. You get home at 7 o'clock at night. You're not thinking healthy … I don't know how many times I did it, and I know it's wrong. Go by Burger King and, you know, give me the dollar menu. Give me 4 hamburgers because I hadn't really had anything to eat all day.^45^ ^,p.158^(United States)

In these examples, participants struggled with their diabetes self‐management due to competing demands on their time and priorities relating to their work and types of occupation. The decisions they made led to their health taking a backseat since other priorities needed to be met such as weighing up which medication they could afford to take and eating for convenience after a long shift.

For those experiencing homelessness, self‐managing their diabetes was no longer a priority:

When I was sitting on a road drinking alcohol, I didn't give a care in the world about my diabetes because I didn't have a home at the time.^33^ ^,p.1037^ (Canada)

There are more serious problems in my life than that [monitoring his blood glucose].^42^ ^,p.123^ (Canada)

When I was homeless, it [diabetes] was very hard to manage it because I would not pick up my prescription. I would leave my bag somewhere because I didn't want to walk around with it. It was just a lot of—it seemed like other things presented itself to be more important than that, so I just overlooked it.^41^ ^,p.74^ (United States)

#### 3.4.6 Health literacy

The quality and quantity of diabetes‐related information that was received by participants was highlighted as being crucial to self‐management. Nearly half of the studies reviewed, highlighted that amongst socioeconomically deprived populations, the level of information provided on diabetes from healthcare professionals and the level of understanding that individuals had about their long‐term condition was a barrier to self‐management:

I did not find the information I received from my doctor as useful. The doctor just wrote something down on a piece of paper and gave it to me.^30^ ^,p.29^ (United States)

They [healthcare provider] just say you have to do that and that and that, but don't tell you how. I need a plan with information on what to eat.^32^ ^,p.7^ (Sweden)

As such, some studies highlighted a need for healthcare professionals to engage more with patients about diabetes self‐management since limited education or understanding posed a further challenge for some participants:

For years, I used to do my blood glucose, but I didn't know what I was doing it for. I just pricked my finger and saw the number. Who was I going to report it to? … I wasn't educated.^23^ ^,p.175^ (United States)

They remind me of things I can eat and cannot eat, I like that. They also give a big paper with that but I don't know how to read, so I always leave it.^46^ ^,p.5^ (Mexico)

Some participants also had misconceptions about diabetes and its treatment, which led to a lack of concordance with medication:

Too much medication makes you sick. The pills themselves turn into rocks in your kidneys, so if the doctor tells me to take three pills, I take two.^46^ ^,p.5^ (Mexico)

Others reported the challenge of communicating with healthcare professionals to improve their health. Challenges ranged from a lack of information provided, inadequate time for appointments and discouraging attitudes from healthcare professionals. One participant explained that while seeking a referral to a podiatrist from her general practitioner (GP), she had to make an additional appointment due to a large amount of paperwork that needed to be completed:

He [doctor] said ‘well it's a lot of work here. There's a lot of paperwork I've got to do’ and it was like ‘okay, I'd better not ask him to do that again’ … I haven't asked the doctor I'm seeing now because Doctor [name] sort of put me off it*….* ^39^,p.340 (Australia)

### 3.5 Facilitators of diabetes self‐management

Diabetes self‐management practices such as self‐monitoring enabled participants to know whether they needed to take any action to manage their diabetes. Eating more fruit and vegetables, reducing the amount of unhealthy food consumed or exercising were all examples of self‐management that appeared in the studies reviewed. These components could be facilitated through different lifestyle choices and having goals, support from healthcare providers, and access to informal support such as peers, family and friends.

#### 3.5.1 Lifestyle and having goals

Self‐management practices amongst those experiencing socioeconomic deprivation were facilitated through building self‐management into everyday life so that it became habitual, and individuals could set achievable goals. Regarding the latter, participating in self‐management interventions which set goals around weight loss or healthy living could result in additional lifestyle changes, greater confidence, and motivation:

I didn't gain this weight overnight, and I'm not going to lose it overnight. [The Diabetes Control Programme] encourages you … It gave me the confidence to be able to go to the gym now and workout and not feel a certain type of way.^23^ ^,p.170^ (United States)

It's not every day but when they send challenges, they helped me a lot. I don't answer them but I read them and I say, ‘I have to do this”. I motivate myself … It makes you think actually about what you're doing to yourself…^24^ ^,p.5^ (Mexico)

#### 3.5.2 Support from healthcare providers

While financial insecurity has been identified as a barrier to diabetes self‐management, eight of the studies highlighted that being in receipt of some form of assistance that helped with their financial circumstances, acted as a facilitator of self‐management. One participant reported that they communicated with their healthcare provider about the socioeconomic challenges they were facing:

When the doctor writes the prescriptions, I let them know that like ‘Hey, I've got $28 I can pay out of my bills’ … and if I've got to have another medication, it's got to be one that my drug coverage can cover.^44^ ^,p.7^ (United States)

The above study reported that by discussing financial hardship and working with their doctor, this participant was able to better manage their medication costs. In another study,^36^ a participant who previously found it difficult to afford test strips and a glucose monitor, was reported to have experienced improvements with her diabetes self‐management once she qualified for Medicaid in the United States. Self‐management could also be facilitated by access to free community services such as health screenings which were valued amongst those with limited financial resources:

At health fairs there is a lot of information available and they give free check‐ups.

We go to health fairs frequently for the same reason, to take advantage of the exams.^43^ ^,p.1093^ (United States)

Another study that looked at adults affected by food insecurity, discussed the practical role that healthcare providers can play in diabetes self‐management, as highlighted in the quote below:

I'd say the most help I get is through this dietitian … she'll tell me … ‘why don't you try this…’ and, you know, I tell her my budget is limited and she'll say, ‘okay, this is cheaper, try that’, and it'd be things I would never think of.^28^ ^,p.6^ (Canada)

The patient–provider relationship was a subtheme that occurred more broadly across the theme of diabetes self‐management facilitators. The topic of being able to build trust and rapport with healthcare providers appeared in nine of the studies. Some participants spoke about the positive impact of being able to connect emotionally with their doctor in relation to managing diabetes,^28^ as well as reporting that they were more likely to act on information that came from a supportive provider.^38^ One participant elaborated on their relationship with a healthcare provider:

I trust the doctor. The nurse that's there is very good. I get treated like a human being, and not just cattle.^37^ ^,p.180^ (Australia)

#### 3.5.3 Informal support

In 18 of the studies, authors discussed the role that informal support can play in diabetes self‐management. Informal support appeared in various guises such as through peer support groups, friends and family. Several studies found that interacting with others who were also diagnosed with diabetes helped with a positive outlook when faced with the challenges of self‐management:

In the community, we don't talk about [living with diabetes]. This class allows us to be able to talk openly about that, and I'm hearing that other people are going through some of the things I'm going through … to hear that other people are dealing with some of the same issues I'm dealing with.^23^ ^,p.176^ (United States)

Because then I can better manage my diabetes with other people in the same situation. We can give each other pointers and help each other out.^37^ ^,p.181^ (Australia)

Participants who had access to peers that were in a relatable situation because they also had diabetes could subsequently engage in information and knowledge exchange which at times was reciprocal.^44^, ^45^ While peers had comparable experiences and encountered similar barriers to self‐management, family members could take on a similar role:

I've been a diabetic for more than 25 years … and I was able to pass on that information to my granddaughters, because diabetes runs in our family, and I was able to explain to them they need to eat better, so they can prevent it.^27^ ^,p.511^ (United States)

Some participants highlighted that family and friends could provide motivation and encouragement which aided self‐management.^23^, ^34^ Others said that family members helped with positive ‘health behaviours’^31^ and with maintaining a healthy diet which provided motivation and encouragement:

It helps if you have someone eating along with you saying don't eat this or don't eat that. My sister encourages me to buy healthy food like I buy wheat noodles instead of regular noodles.^44^ ^,p.7^ (United States)

They [family] try to help me with everything. My wife is trying to help me with the cooking and such. My wife watches what I eat. She makes a lot of salad, fish, chicken and little rice and no sugar.^32^ ^,p.9^ (Sweden)

## 4 DISCUSSION

This systematic review has identified key barriers and facilitators of self‐management of diabetes type 1 and 2 amongst people who are socioeconomically deprived. The findings support evidence that self‐management interventions can be less effective in socioeconomically deprived populations.^7^ While other reviews have focused on the relationship between self‐efficacy and diabetes self‐management, this review has included underexplored factors such as ‘social support, financial issues, and access to health care’.^50^ Access to medication, health services and health information needed to facilitate self‐management pose a challenge to individuals living in countries such as the United States where there is no universal health system. Further barriers include the financial cost of healthy eating, which is important when self‐managing diabetes, as well as a lack of culturally appropriate dietary suggestions for those looking to make changes to their diet. Living in an area of deprivation meant services were stretched and therefore limited and could also cause a feeling of unsafety, leading to people not wanting to leave their home to exercise or access services. Many of the participants were of working age but were employed in jobs with little autonomy, meaning they could not manage their diabetes effectively. Despite these barriers, participants were resilient and found setting goals and accessing healthcare and informal support key to diabetes self‐management.

The studies in the primary papers in this systematic review were mainly conducted in the United States. Previous research from the United States shows that community characteristics, which relate to features within the local area where people live, along with individual sociodemographic characteristics (e.g., age, gender, race/ethnicity, educational attainment, employment status) can limit people's access to medical care.^51^ A lack of financial resources amongst a population largely reliant upon healthcare to assist with diabetes self‐management^8^ and lack of awareness amongst healthcare professionals of the socioeconomic challenges are subsequently compounded by structural factors surrounding health systems, leading to health inequalities.

As this review has indicated, low medication adherence amongst people living with diabetes in the United States is associated with the ‘high cost of medications, especially the injectable medications’ that are not covered by insurance.^52^ Barriers associated with medication adherence can also arise in countries where there is access to a universal health system. While people diagnosed with diabetes in the United Kingdom are entitled to an exemption certificate to get free prescriptions under the National Health Service, which assists with overcoming some financial barriers, there are additional barriers which also impact on diabetes self‐management. For example, amongst those with low health literacy, especially low numeracy, difficulties can be experienced due to the mathematics involved for individuals who are trying to manage their own insulin doses.^53^

The socioeconomic challenges associated with self‐management of diabetes therefore goes beyond the cost of healthcare and medication. Many participants described a lack of trust in healthcare providers as well as general confusion about diabetes medications and management. Health literacy concerns ‘a person's knowledge, motivation and competencies to access, understand, appraise, and apply health information in order to make judgements and take decisions in everyday life concerning healthcare, disease prevention and health promotion to maintain or improve quality of life during the life course’.^54^ Therefore, health literacy barriers can arise from a lack of clear health information, as well as poor literacy or numeracy skills. A systematic review on the prevalence of limited health literacy amongst patients with diabetes suggests this is linked to low levels of diabetes‐specific knowledge, struggles with patient–provider communications and understanding of medical terminology.^55^ Literature shows a correlation between low or inadequate health literacy and populations that are socioeconomically deprived.^56^, ^57^ This review highlights a need, therefore, for improved engagement between diabetes patients and health services, including greater awareness of the cultural and socioeconomic barriers of diabetes self‐management.

This review illustrates the sacrifices that people living with diabetes face due to financial insecurity more broadly. As well as foregoing essential medications, many people can struggle to afford the food needed to maintain a healthy diet. Food insecurity (or food poverty) which is closely associated with financial insecurity, ‘affects adherence to dietary and self‐care behaviours, including blood glucose monitoring’ and has subsequently been associated with poor glycaemic control amongst people with diabetes.^58^ Food insecurity is a major risk factor for people with diabetes^59^ and there is a burgeoning body of literature on food insecurity across high‐income countries including the United States, Canada, Germany and the United Kingdom.^60^, ^61^, ^62^, ^63^, ^64^ Food bank use is associated with food insecurity and research from the United Kingdom shows that nearly 75% of food bank users had at least one health issue.^65^ There is evidence to suggest that food bank intervention activities, which focus on distributing diabetes‐appropriate food packages for improving diet, can have a positive impact on decreasing the consumption of unhealthy foods significantly.^59^

Additionally, eating a diet to support self‐management of diabetes can be further hindered due to a lack of culturally appropriate food suggestions for people from ethnic minority groups living with diabetes. Norms surrounding individual behaviours that are associated with culture and class may also act in opposition to making positive health choices around diet.^66^ For instance, alcohol consumption is identified as an important aspect amongst some working‐class men but is at odds with diabetes self‐management.^39^ Gendered social networks can however have a strong influence over health behaviours and pose a challenge to health compliance.^39^ As such, while social networks made up of peers in a relatable position are shown to be beneficial for people living with diabetes, informal support that is built around reciprocity is needed to help mobilise solidarity.^67^

Although there were many challenges to self‐management of diabetes, there were also many facilitators. Building trusting and consistent relationships with healthcare providers was a key facilitator to self‐management. People with diabetes are shown to benefit from access to healthcare providers that have an awareness of the challenges facing socioeconomically deprived populations.^44^ As mentioned, peer support also helps facilitate self‐management since being around others who have the same condition is beneficial. Peer support through contact with relatable people assisted with a positive outlook and enabled reciprocity through information sharing and knowledge exchange based on personal experiences of self‐management practices. Peer support can be in the form of informal support, for example, through a friend or family member or through a group to facilitate peer‐based interventions. The latter is a common method which is found to be effective for enforcing positive health behaviours since they are shown to help promote and share specific health messages and improve self‐care and self‐efficacy.^68^, ^69^ Setting goals also led to positive health behaviours, resulting in lifestyle changes, greater confidence, and motivation relating to diabetes self‐management. Research suggests that assisting people to achieve diabetes goals which focus on improvement to lifestyle, overall quality of life, and psychological well‐being may, in the long‐term, be more effective than focusing on diabetes outcomes.^70^

## 5 STRENGTHS AND LIMITATIONS OF THE REVIEW

This review has provided an international perspective on the topic of diabetes self‐management, focusing on identifying the facilitators and barriers amongst socioeconomically deprived populations. The participant samples included people from ethnically diverse backgrounds and thus provided some insights around the cultural challenges that people with diabetes face, both at home and within families. While providing an international perspective on diabetes self‐management amongst people experiencing socioeconomic deprivation is beneficial for understanding the structural barriers associated with different health systems, the review identified few European studies and does not include any UK‐based studies. Additionally, whilst the review included type 1 and 2 diabetes, most of the data focused on the management of type 2 diabetes meaning there may be additional implications for type 1 that have not been explored in this review.

## 6 IMPLICATIONS FOR CLINICAL PRACTICE AND FUTURE RESEARCH

The review highlights the need for more European and UK‐based studies to understand how individuals with diabetes, who also experience socioeconomic deprivation, manage their conditions in these contexts. There is evidence to suggest that the structural barriers surrounding health systems play a large part in creating challenges around diabetes self‐management and future research could explore the how health providers support people experiencing socioeconomic deprivation to self‐manage their diabetes. Self‐management interventions need to be affordable as well as inclusive from a cultural perspective. Co‐design may support the development of information that is culturally appropriate and easy to access and understand. In clinical practice, healthcare providers/professionals can be supported through cultural competence training to increase cultural awareness in healthcare and ensure patients have access to the appropriate support and information needed to help them self‐manage.

## 7 CONCLUSION

This review has highlighted many barriers and facilitators to self‐management of diabetes in socioeconomically deprived populations. Many of these barriers, such as living in areas of deprivation and financial barriers to healthcare, medication and healthy food requires structural and policy‐level changes. However, other barriers such as providing clear, culturally appropriate health information and facilitators such as support setting goals can be developed in the form of self‐management interventions. Supporting people who are experiencing socioeconomic deprivation to self‐manage diabetes, as well as other long‐term conditions, is key to reducing health inequalities.

## AUTHOR CONTRIBUTIONS

**Abi Woodward**: Writing—original draft; writing—review and editing; formal analysis; methodology; data curation; conceptualisation. **Kate Walters**: Writing—review and editing. **Nathan Davies**: Writing—review and editing. **Danielle Nimmons**: Writing—review and editing. **Joanne Protheroe**: Writing—review and editing. **Carolyn A Chew‐Graham**: Writing—review and editing. **Fiona Stevenson**: Writing—review and editing. **Megan Armstrong**: Writing—review and editing; supervision; funding acquisition.

## CONFLICT OF INTEREST STATEMENT

Professor Carolyn A. Chew‐Graham is Editor in Chief of Health Expectations. The remaining authors declare no conflict of interest.

## Supporting information

Supporting information.

Supporting information.

Supporting information.

## Data Availability

Data sharing is not applicable to this article as no new data were created or analysed in this study.
