# Supplementary material for: Barriers and facilitators of self‐management of diabetes amongst people experiencing socioeconomic deprivation: A systematic review and qualitative synthesis
Source: Health Expect. 2024 May 15;27(3):e14070. doi: 10.1111/hex.14070 (PMC11096776; doi:10.1111/hex.14070)
Supplement: Supplementary file 2 — Supporting information. [file HEX-27-e14070-s002.docx]

| **Source Paper** | **Title of Paper** | **CASP checklist: Quality Assessment** | | | | | | | | | |
| --- | --- | --- | --- | --- | --- | --- | --- | --- | --- | --- | --- |
|  |  | Was there a clear statement of the aims of the research? | Is a qualitative methodology appropriate? | Was the research design appropriate to address the aims of the research? | Was the recruitment strategy appropriate to the aims of the research? | Was the data collected in a way that addressed the research issue? | Has the relationship between researcher and participants been adequately considered? | Have ethical issues been taken into consideration? | Was the data analysis sufficiently rigorous? | Is there a clear statement of findings? | How valuable is the research? |
| Akohhoue et al., 2015, United States | Patients', Caregivers', and Providers' Perceived Strategies for Diabetes Care | Yes | Yes | Yes | Yes | Yes | Yes | Yes | Can't tell | No | Contributes to knowledge about strategies to improve self-management and how interventions could address impact of strategies/barriers to help low-income and minority patients to better manage their diabetes. |
| Allen et al., 2020, United States | Engaging Underserved Community Members in Diabetes Self-Management: Evidence From the YMCA of Greater Richmond Diabetes Control Program | Yes | Yes | Yes | Yes | Yes | Yes | Yes | Yes | Yes | Contributes to existing knowledge on how to encourage participant engagement in diabetes self-management programmes and identifies multiple actionable recommendations for enhancing engagement of traditionally underserved groups in diabetes care. |
| Aweko et al., 2018, Sweden | Patient and Provider Dilemmas of Type 2 Diabetes Self-Management: A Qualitative Study in Socioeconomically Disadvantaged Communities in Stockholm | Yes | Yes | Yes | Yes | Yes | Yes | Yes | Yes | Yes | Research highlights the dilemmas encountered by patients and providers when considering patient self-management which could help inform future efforts to assist patients better manage their T2DM. |
| Burner et al., 2014, United States | Perceptions of Successful Cues to Action and Opportunities to Augment Behavioural Triggers in Diabetes Self-Management: Qualitative Analysis of a Mobile Intervention for Low-Income Latinos With Diabetes | Yes | Yes | Yes | Yes | Yes | Yes | Yes | Yes | Yes | Valuable as provides insight into how and why mHealth intervention functioned, allowing for the future development of more effective interventions. Further research should investigate whether personalisation results in improved health outcomes. |
| Campbell et al., 2020, Canada | The Challenges of Managing Diabetes While Homeless: A Qualitative Study Using Photovoice Methodology | Yes | Yes | Yes | Yes | Yes | Yes | Yes | Yes | Yes | Valuable insight into the challenges of diabetes management faced by people experiencing homelessness which could assist providers and policy-makers to better meet the needs of this population. |
| Carolan et al., 2014, Australia | Experiences of Diabetes Self-Management: A Focus Group Study Among Australians With Type 2 Diabetes | Yes | Yes | Yes | Yes | Yes | Yes | Yes | Yes | Yes | Research is valuable as it reveals a number of unmet information and support needs for individuals with type 2 diabetes mellitus - potential to lead to improvements in experience and self-management outcomes for individuals with T2DM. |
| Chan et al., 2015, Canada | Challenges of Diabetes Self-Management in Adults Affected by Food Insecurity in a Large Urban Centre of Ontario, Canada | Yes | Yes | Yes | Yes | Yes | Can't tell | Yes | Yes | Yes | Valuable insights for health care providers regarding the tailoring of diabetes management plans and community programs within the context of food insecurity. |
| Christensen et al., 2020, Denmark | A Qualitative Exploration of Facilitators and Barriers for Diabetes Self-Management Behaviours Among Persons with Type 2 Diabetes from a Socially Disadvantaged Area | Yes | Yes | Yes | Yes | Yes | Yes | Yes | Yes | Yes | Somewhat valuable as provides insight into barriers/facilitators of diabetes self-management and need for tailored healthcare. Research is limited due to a relatively small body of empirical data. |
| Clark et al., 2009, United States | Cultural Values and Political Economic Contexts of Diabetes Among Low-Income Mexican Americans | Yes | Yes | Yes | Yes | Yes | Yes | Yes | Yes | Yes | Somewhat valuable as provides insight into how culture shapes diabetes self-management in a low socioeconomic environment which has implications for practice. |
| Dao et al., 2019, Australia | Factors Influencing Self-Management in Patients with Type 2 Diabetes in General Practice: A Qualitative Study | Yes | Yes | Yes | Yes | Yes | Yes | Yes | Yes | Yes | Somewhat valuable as suggests strategies to improve patient self-management Recommends that multi-level interventions could be used to systematically target areas of change at each level of influence |
| Fritz, 2015, United States | Learning to do Better: The Transactional Model of Diabetes Self-Management Integration | Yes | Yes | Yes | Yes | Yes | Yes | Yes | Yes | Yes | Findings contribute new understandings regarding why some people might initiate components of Diabetes Self-management yet fail to truly incorporate them into their daily life. Limited however due to relatively small sample and geographically limited urban context. |
| Gazmararian et al., 2009, United States | Perception of Barriers to Self-Care Management Among Diabetic Patient | Yes | Yes | Yes | Yes | Yes | Yes | Yes | Yes | Yes | Somewhat valuable as results may be useful for improving the delivery of care and to develop quantitative studies to explore particular areas of interest. |
| Henderson et al., 2014, Australia | Social Barriers to Type 2 Diabetes Self-Management: The Role of Capital | Yes | Yes | Yes | Yes | Yes | Yes | Yes | Yes | Yes | Somewhat valuable to policy and practice. Highlights that poor access to social and cultural capital may be preventing participants from engaging with health professionals as partners in care. |
| Hu et al., 2013, United States | Perceptions of Barriers in Managing Diabetes: Perspectives of Hispanic Immigrant Patients and Family Members | Yes | Yes | Yes | Yes | Yes | Yes | Yes | Yes | Yes | Valuable research for improving diabetes outcomes and reducing diabetes complications among an ethnically diverse population. Provides evidence base for further research into barriers and effective intervention. |
| Keene et al., 2018, United States | "That wasn't really a place to worry about diabetes": Housing Access and Diabetes Self-Management Among Low-Income Adults | Yes | Yes | Yes | Yes | Yes | Can't tell | Yes | Yes | Yes | Somewhat valuable. Provides contribution to research on how improved affordable housing access may represent an opportunity to improve outcomes and reduce socioeconomic disparities among those living with type 2 diabetes. |
| Luo & White-Means, 2021, United States | Evaluating the Potential Use of Smartphone Apps for Diabetes Self-Management in an Underserved Population: A Qualitative Approach | Yes | Yes | Yes | Yes | Yes | Yes | Yes | Yes | Yes | Addressed a gap in the literature regarding mHealth apps addressing diabetes-related health disparities and emphasised need to engage underserved groups with mHealth. |
| Lynch et al., 2021, United States | Concepts of Diabetes Self-Management in Mexican American and African American Low-Income Patients With Diabetes | Yes | Yes | Yes | Yes | Yes | Yes | Yes | Yes | Yes | Contributes to knowledge around the use of diabetes self-management among ethnic groups. The findings are limited to African Americans and Mexican Americans but the hypothesis generated in the study provides potential to inform future studies with a more diverse population. |
| Mamykina et al., 2016, United States | Structured Scaffolding for Reflection and Problem Solving in Diabetes Self-Management: Qualitative Study of Mobile Diabetes Detective | Yes | Yes | Yes | Yes | Yes | Yes | Can't tell | Yes | Yes | Valuable as highlights importance of participants' ability to notice and understand trends in data about their condition. Future development of informatic interventions could facilitate self-management. Limitations to the study i.e., reliance on subject reports from participants were, at the time being addressed through an ongoing randomised control trial. |
| Onwudiwe et al., 2011, United States | Barriers to Self-Management of Diabetes: A Qualitative Study Among Low-Income Minority Diabetics | Yes | Yes | Yes | Yes | Yes | No | Yes | Can't tell | Yes | Provides useful insights around how patients' limited health literacy acts as a barrier to self-management. Includes suggestions for future diabetes management interventions and how physicians can facilitate patients' self-management but these could be more explicit. |
| Pilkington et al., 2010, Canada | The Experience of Living with Diabetes for Low-Income Canadians | Yes | Yes | Yes | Yes | Yes | Yes | Yes | Yes | Yes | Adds to relevant literature on how individuals learn to self-manage diabetes through experience and experimentation. Provides new knowledge through understanding the additional implications of living on a low-income, raising questions around how socioeconomic deprivation interacts with health teaching/knowledge. |
| Ramal et al., 2012, United States | Factors that Influence Diabetes Self-Management in Hispanics Living in Low Socioeconomic Neighbourhoods in San Bernadino, California | Yes | Yes | Yes | Yes | Yes | Yes | Yes | Yes | Yes | Somewhat valuable it highlights the need to increase patient awareness of available community resources. Limited by sample size and further research is needed to explore strategies enhancing self-efficacy in regards to increasing health. |
| Reyes et al., 2017, United States | Factors Influencing Diabetes Self-Management Among Medically Underserved Patients With Type II Diabetes | Yes | Yes | Yes | Yes | Yes | Yes | Yes | Yes | Yes | Contributes new research around patient perceived control of self-management between those with good and poor diabetes control. Implications for providers and interventions to provide formal and informal support, address health literacy and mental health. |
| Shepherd-Banigan et al., 2014, United States | "The Promotora Explained Everything" Participant Experiences During a Household-Level Diabetes Education Program | Yes | Yes | Yes | Yes | Yes | Yes | Yes | Yes | Yes | Somewhat valuable as supports prior findings that culturally tailored interventions are more effective in achieving positive change . Provides new insight into mechanisms that promote behaviour change for underserved Hispanic populations. |
| Stotz et al., 2021, United States | Opportunities for Interventions That Address Socioeconomic Barriers to Type 2 Diabetes Management: Patient Perspectives | Yes | Yes | Yes | Yes | Yes | Can't tell | Yes | Yes | Yes | Valuable as builds on prior research on patient perspectives on developing interventions addressing social determinants of health to increase diabetes self-management. Limited by sample. |
| Vest et al., 2013, United States | Diabetes Self-Management In A Low-Income Population: Impacts of Social Support and Relationships With the Health Care System | Yes | Yes | Yes | Yes | Yes | Yes | Yes | Yes | Yes | Identifies a need for future research to take a broader approach to exploring diabetes self-management to go beyond individual patient behaviour and clinical guidelines. Recommendations put forward for clinical practice to take individual, social and cultural factors into account in order to improve health. |
| Whittemore et al., 2019, Mexico | Challenges to Diabetes Self-Management For Adults With Type 2 Diabetes in Low-Resource Settings in Mexico City: A Qualitative Descriptive Study | Yes | Yes | Yes | Yes | Yes | Yes | Yes | Yes | Yes | Builds upon previous literature regarding challenges of accessing diabetes self-management education and services but further research needed on how to implement patient-centred diabetes prevention and self-management programs for underserved groups. |
