# Supplementary material for: Barriers and facilitators of self‐management of diabetes amongst people experiencing socioeconomic deprivation: A systematic review and qualitative synthesis
Source: Health Expect. 2024 May 15;27(3):e14070. doi: 10.1111/hex.14070 (PMC11096776; doi:10.1111/hex.14070)
Supplement: Supplementary file 3 — Supporting information. [file HEX-27-e14070-s001.docx]

| **CERQual Qualitative Evidence Assessment** | | | | | | | |
| --- | --- | --- | --- | --- | --- | --- | --- |
| **Review finding** | **Studies contributing to review finding** | **Assessment of methodological limitations** | **Assessment of relevance** | **Assessment of coherence** | **Assessment of adequacy** | **Overall CerQual assessment of confidence** | **Explanation of judgment** |
| Healthcare costs as a socioeconomic barrier to diabetes self-management | Keene, D.E., et al., 2018. Campbell, R.B., et al., 2021.  Clark, L., et al., 2009.  Henderson, J., et al., 2014.  Hu, J., et al., 2013.  Lynch, E., et al.,2012.  Pilkington, F.B., et al., 2010.  Reyes, J., et al., 2017.  Whittemore, R., et al., 2019.  Vest, B.M., et al., 2013. | No or very minor concerns (all papers were found to have used appropriate methodology with no limitations) | Minor concerns about relevance (no studies were from the UK) | Minor concerns about coherence (data reasonably consistent within and across all studies) | No or very minor concerns about adequacy (10 papers offered rich data) | High confidence | This finding was graded as high  confidence because of minor  concerns regarding  methodological limitations,  relevance, coherence, and  adequacy. |
| Financial costs of healthy eating as a socioeconomic barrier to diabetes self-management | Clark, L., et al., 2009.  Pilkington, F.B., et al., 2010.  Reyes, J., et al., 2017.  Stotz, S.A., et al., 2021.  Chan, J., et al., 2015.  Aweko, J., et al., 2018 | No or very minor concerns (2 papers were assessed as having methodological limitations concerning reflexivity i.e. the relationship between data collectors and participants) | Minor concerns about relevance (no studies were from the UK) | Minor concerns about coherence (data reasonably consistent within and across all studies) | No or very minor concerns about adequacy (6 papers offered rich data) | High confidence | This finding was graded as high  confidence because of minor  concerns regarding  methodological limitations,  relevance, coherence, and  adequacy. |
| Cultural influences as a socioeconomic barrier to diabetes self-management | Henderson, J., et al., 2014.  Pilkington, F.B., et al., 2010. | No or very minor concerns (all papers were found to have used appropriate methodology with no limitations) | Minor concerns about relevance (no studies were from the UK) | Minor concerns about coherence (data reasonably consistent within and across all studies) | Minor concerns about adequacy (2 papers offered rich data) | Moderate confidence | This finding was graded as moderate confidence because of minor concerns regarding relevance, coherence and adequacy. |
| Living in areas of deprivation as a socioeconomic barrier to diabetes self-management | Keene, D.E., et al., 2018.  Campbell, R.B., et al., 2021.  Henderson, J., et al., 2014.  Pilkington, F.B., et al., 2010.  Christensen, N.I., et al., 2020. | No or very minor concerns (1 paper was assessed as having methodological limitations concerning reflexivity i.e. the relationship between data collectors and participants) | Minor concerns about relevance (no studies were from the UK) | Minor concerns about coherence (data reasonably consistent within and across all studies) | No or very minor concerns about adequacy (5 papers offered rich data) | High confidence | This finding was graded as high  confidence because of minor  concerns regarding  methodological limitations,  relevance, coherence, and  adequacy. |
| Competing priorities and time constraints as a socioeconomic barrier to diabetes self-management | Keene, D.E., et al., 2018.  Pilkington, F.B., et al., 2010.  Reyes, J., et al., 2017.  Whittemore, R., et al., 2019.  Stotz, S.A., et al., 2021.  Gazmararian, J.A., et al., 2009.  Dao, J., et al., 2019.  Fritz, H.A., 2015. | No or very minor concerns (2 papers were assessed as having methodological limitations concerning reflexivity i.e. the relationship between data collectors and participants) | Minor concerns about relevance (no studies were from the UK) | Minor concerns about coherence (data reasonably consistent within and across all studies) | No or very minor concerns about adequacy (9 papers offered rich data) | High confidence | This finding was graded as high  confidence because of minor  concerns regarding  methodological limitations,  relevance, coherence, and  adequacy. |
| Health literacy as a socioeconomic barrier to diabetes self-management | Henderson, J., et al., 2014.  Whittemore, R., et al., 2019  Aweko, J., et al., 2018.  Onwudiwe, N.C., et al., 2011.  Allen, J.O., et al., 2020. | No or very minor concerns 1 paper was assessed as having methodological limitations concerning the sufficiency of detail provided around data analysis) | Minor concerns about relevance (no studies were from the UK) | Minor concerns about coherence (data reasonably consistent within and across all studies) | No or very minor concerns about adequacy (5 papers offered rich data) | High confidence | This finding was graded as high  confidence because of minor  concerns regarding  methodological limitations,  relevance, coherence, and  adequacy. |
| Lifestyle and goals as a facilitator of diabetes self-management | Allen, J.O., et al., 2020.  Burner, E.R., et al., 2014. | No or very minor concerns (all papers were found to have used appropriate methodology with no limitations) | Minor concerns about relevance (no studies were from the UK) | Minor concerns about coherence (data reasonably consistent within and across all studies) | Minor concerns about adequacy (2 papers offered rich data | Moderate confidence | This finding was graded as moderate confidence because of minor concerns regarding relevance, coherence and adequacy |
| Support from healthcare professionals as a facilitator of diabetes self-management | Clark, L., et al., 2009.  Reyes, J., et al., 2017.  Chan, J., et al., 2015.  Dao, J., et al., 2019.  Fritz, H.A., 2015.  Ramal, E., et al., 2012. | No or very minor concerns (1 paper was assessed as having methodological limitations concerning reflexivity i.e. the relationship between data collectors and participants) | Minor concerns about relevance (no studies were from the UK) | Minor concerns about coherence (data reasonably consistent within and across all studies) | No or very minor concerns about adequacy (6 papers offered rich data) | High confidence | This finding was graded as high  confidence because of minor  concerns regarding  methodological limitations,  relevance, coherence, and  adequacy. |
| Informal support as a facilitator of diabetes self-management | [1-8]Reyes, J., et al., 2017.  Vest, B.M., et al., 2013.  Stotz, S.A., et al., 2021.  Aweko, J., et al., 2018.  Dao, J., et al., 2019.  Allen, J.O., et al., 2020.  Shepherd-Banigan, M., et al., 2014.  Carolan, M., J., et al., 2015. | No or very minor concerns (1 paper was assessed as having methodological limitations concerning reflexivity i.e. the relationship between data collectors and participants) | Minor concerns about relevance (no studies were from the UK) | Minor concerns about coherence (data reasonably consistent within and across all studies) | No or very minor concerns about adequacy (8 papers offered rich data) | High confidence | This finding was graded as high  confidence because of minor  concerns regarding  methodological limitations,  relevance, coherence, and  adequacy. |
